# Revealing the constituents of Egypt’s oldest beer using infrared and mass spectrometry

**DOI:** 10.1038/s41598-019-52877-0

**Published:** 2019-11-07

**Authors:** Mohamed A. Farag, Moamen M. Elmassry, Masahiro Baba, Renée Friedman

**Affiliations:** 1 Pharmacognosy Department, Faculty of Pharmacy, Cairo University, Cairo, 11562 Egypt; 2 Departmenst of Chemistry, School of Sciences & Engineering, The American University in Cairo, New Cairo, 11835 Egypt; 3 Department of Biological Sciences, Texas Tech University, Lubbock, TX USA; 4 Waseda Institute for Advanced Study, Waseda University, Tokyo, Japan; 5 The Griffith Institute, University of Oxford, Oxford, UK

**Keywords:** Metabolomics, Mass spectrometry, Gas chromatography, Solid-phase microextraction, Infrared spectroscopy

## Abstract

Previous studies have shown that the Ancient Egyptians used malted wheat and barley as the main ingredients in beer brewing, but the chemical determination of the exact recipe is still lacking. To investigate the constituents of ancient beer, we conducted a detailed IR and GC-MS based metabolite analyses targeting volatile and non-volatile metabolites on the residues recovered from the interior of vats in what is currently the world’s oldest (c. 3600 BCE) installation for large-scale beer production located at the major pre-pharaonic political center at Hierakonpolis, Egypt. In addition to distinguishing the chemical signatures of various flavoring agents, such as dates, a significant result of our analysis is the finding, for the first time, of phosphoric acid in high level probably used as a preservative much like in modern beverages. This suggests that the early brewers had acquired the knowledge needed to efficiently produce and preserve large quantities of beer. This study provides the most detailed chemical profile of an ancient beer using modern spectrometric techniques and providing evidence for the likely starting materials used in beer brewing.

## Introduction

**Figure 1:**
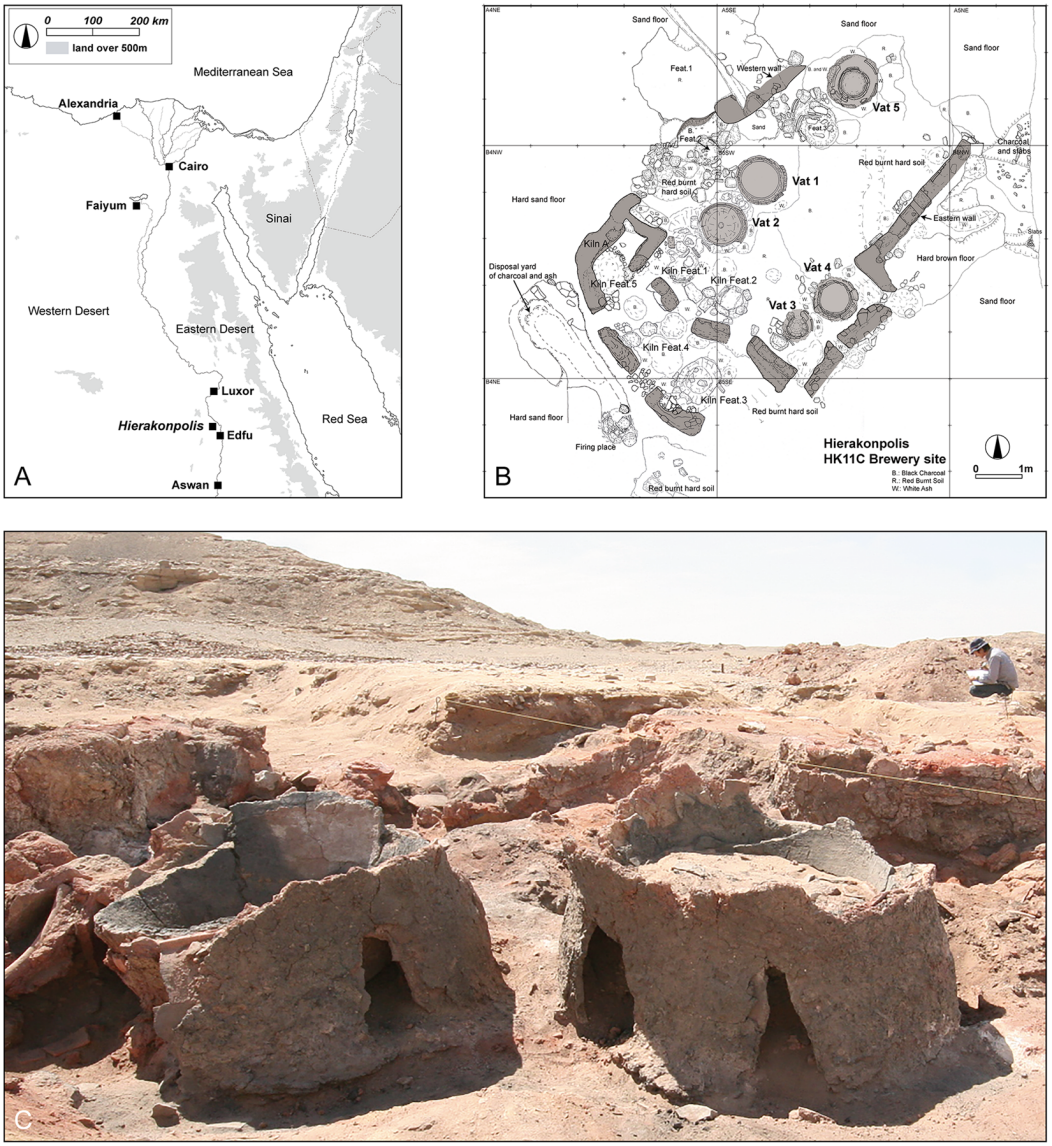
The excavation site at locality HK11C, Hierakonpolis. (**A**) General map of Egypt showing location of Hierakonpolis; (**B**) Plan of the heating installation; (**C**) Vats 1 and 2 viewed from the northeast. (**A**,**B**) created by Masahiro Baba using Adobe Illustrator; (**C**) photo by Masahiro Baba.

Together with bread, beer was considered a staple food for the ancient Egyptians. Moreover, it was an essential provision for their afterlife as shown by numerous depictions and models of brewing found in their tombs. The archaeological record indicates that beer production dates back before the pharaoh, to the Predynastic period (4000-3100 BCE (Before Common Era)), which is the formative stage of the Egyptian civilization. Heating installations related to beer production involving large ceramic vats (serving as mashtuns) have been reported at several sites of this era^1,2^, with a significant number detected by excavation and remote sensing at the major population center of ancient Hierakonpolis^3^. Hierakonpolis is located on the west bank of the Nile, 17 km north of the modern town of Edfu in Upper Egypt (Fig. 1A). Stretching for over 3 km along the Nile, it is one of the largest sites of the Predynastic period^3^. Excavations in a part of the site known as locality HK11C revealed a well-preserved brewery establishment consisting of five freestanding ceramic vats placed beside low wall segments (Fig. 1B)^4^. Only the lower parts of the vats are preserved, but the remains still stand to a height of 40 to 60 cm with diameters ranging from 60 to 85 cm (Fig. 1C). The vat exteriors had been coated with mud and pottery sherds to protect from thermal shock and promote even heating, and a ring of large sherds cemented together with mud placed around the base aided stability and enclosed the fire. Rubification of the construction materials and the high amount of charcoal and ash in the surroundings leave no doubt that these vats functioned as heating installations.

Adhering to the interior of each vat was a thick layer (up to 3 cm) of residue with a shiny black skin. Botanical examinations of the residue indicated that the main ingredients were emmer wheat with some barley. These grains were coarsely crushed or ground and then heated in water together with grains that had been malted in their husks^5^. In addition, the scanning electron microscopy analysis revealed the presence of starch grains with perforation suggestive of enzymatic action prior to fermentation^5^. These findings indicate that the vats served to prepare the wort, the final product being beer. Radiocarbon testing of a sample of residue provides a date of 3764-3537 calibrated BCE (2 sigma) (^14^C 4875 ± 40 Before Present (BP))_,_ make this installation the oldest dated brewery in Egypt. Calculating from the size and number of the vats, a total of 325 liters of beer could have been produced at one time. This quantity, roughly equivalent to 650 modern bottles, indicates a scale of production, undocumented elsewhere in the world at this time. Moreover, integrated into the brewing facility were several pottery kilns to fire the jars that contained the brew (Fig. 1B).

Herein, our chemical analysis aims to provide insight on the brewing method and the product composition of this ancient beer. To comprehensively assess the constituents of the solid residue derived from the vats, we utilized a sensitive mass spectrometric approach. Four samples from two vats were subjected to analysis for their volatile components, directly using solid phase microextraction (SPME) coupled to gas chromatography-mass spectrometry (GC-MS) versus analysis of non-volatile primary metabolites *viz*., sugars, amino acids and organic acids using GC-MS post-silylation.

## Results and Discussion

### GC-MS and IR analysis of primary metabolites in the beer residue

**Figure 2:**
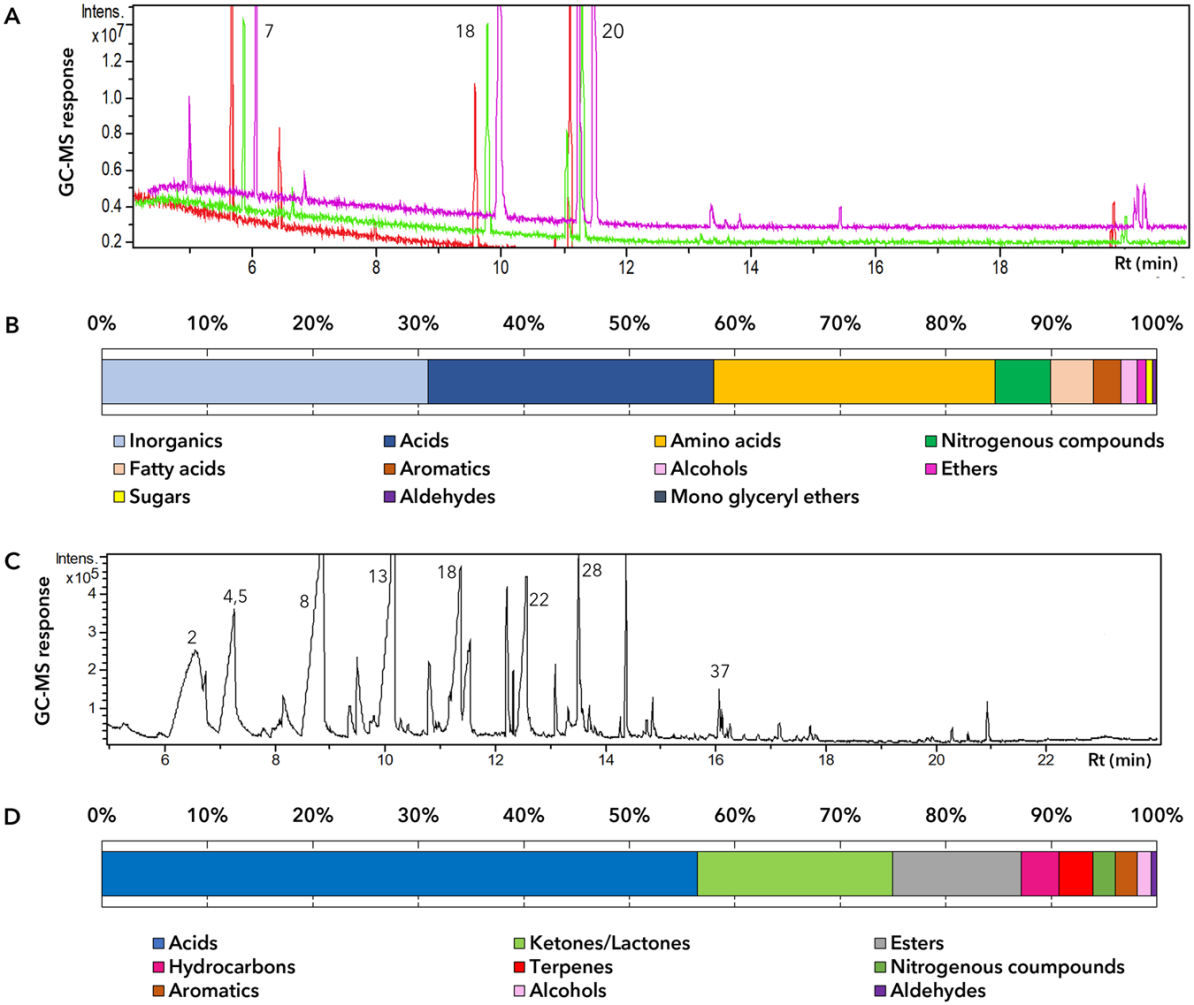
Metabolites analysis of the beer resides from the vats. (**A**) Representative overlaid chromatograms of non-volatile primary metabolites in vats analyzed as silylated products; (**B**) Relative percentage of chemical classes of compounds in beer residue samples as analyzed using GC-MS for non-volatiles analysis post silylation; (**C**) Representative GC-MS chromatogram of headspace volatiles collected from vats; (**D**) Relative percentage of chemical classes of volatile compounds in beer residue samples as analyzed using GC-MS.

**Figure 3:**
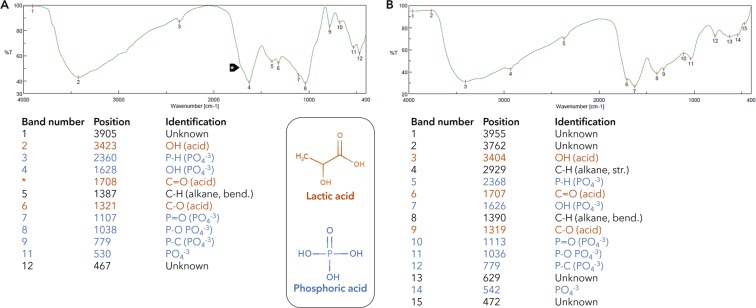
IR spectra from beer residue vat 1 (**A**) and vat 2 (**B**) showing IR absorption bands indicative of organic and phosphoric acid metabolites.

45 Compounds belonging to 11 chemical classes of metabolites were identified in the beer residue. Most abundant were inorganic (31.7%), acids (27.8%), and amino acids (27.4%) (Fig. 2A,B, Table 1). Other chemical classes comprised nitrogenous compounds, fatty acids, aromatics, alcohols, ethers, sugars, aldehydes, and mono glyceryl ethers, although these were present at much lower levels. Phosphoric acid, one of the detected inorganics, was found at high levels (26.7%) in comparison with other identified metabolites in all samples. This was confirmed using infrared (IR) analysis that showed bands indicative of phosphoric acid (Fig. 2). These bands corresponded to the following functional groups and their wave numbers: P-H (PO_4_^−3^), 2360–2368 cm^−1^; OH (PO_4_^−3^), 1625–1627 cm^−1^; P = O (PO_4_^−3^), 1106 cm^−1^; P-O (PO_4_^−3^), 1035–1037 cm^−1^; P-C (PO_4_^−3^), 779 cm^−1^; and PO_4_^−3^, 530–541 cm^−1^ (Fig. 3). Today phosphoric acid is a common additive to alcoholic and non-alcoholic beverages and is employed to prolong their shelf life and enhance flavor^6–8^. This is the first time such a high abundance of phosphoric acid has been detected in ancient Egyptian beer^9^. The source of phosphoric acid is yet to be determined conclusively. However, it is likely that barley grains, which are rich in readily soluble phosphoric acid, are the source for this acid in the beer residue^10,11^. It is possible that the ancient Egyptians utilized the phosphoric acid in barley as a food preservation, much in the same as hops is currently used to impart a flavor and for its preservative effect during beer fermentation^12^. Previously, the earliest evidence of phosphoric acid was from two tripod cooking pots from Crete dating to around 1700 BCE where it was found together with dimethyl oxalate, a basic constituent of modern beer^13^.

**Table 1:** Relative percentage of silylated compounds detected in beer residues from vats using GC-MS for primary metabolites analysis (n = 4).

Peak	Rt (min)	RI	Compound Name	Class	Average (Standard Deviation)
1	4.65	1030	Lactic acid^a^	Acid	3.08 (0.23)
2	4.66	1031	α-Hydroxyisobutyric acid	Acid	0.72 (0.32)
3	4.66	1033	Malonic acid	Acid	3.08 (0.23)
4	5.05	1041	Acetic acid	Acid	0.63 (0.12)
5	5.08	1043	Hexanoic acid	Acid	0.09 (0)
8	6.49	1085	Oxalic acid^a^	Acid	4.68 (2.96)
10	6.87	1095	β-Hydroxybutyric acid	Acid	0.02 (0.01)
11	7.24	1105	Heptanoic acid	Acid	0.06 (0.01)
14	8.07	1130	Hydroxyisovaleric acid	Acid	0.08 (0.04)
16	9.34	1167	Benzoic acid^a^	Acid	0.60 (0.36)
19	10.88	1207	Succinic acid^a^	Acid	7.38 (3.19)
21	11.12	1214	Methyl succinic acid	Acid	1.38 (0.25)
22	12.07	1236	Nonanoic acid	Acid	0.02 (0.01)
23	12.37	1243	Citramalic acid	Acid	0.56 (0.31)
25	13.23	1265	Glutaric acid	Acid	0.85 (0.27)
27	13.68	1276	Methylglutaric acid	Acid	0.29 (0.14)
28	14.49	1295	Citramalic acid	Acid	0.06 (0.03)
29	15.06	1309	Malic acid^a^	Acid	0.38 (0.23)
30	15.69	1323	Adipic acid	Acid	0.21 (0.15)
33	18	1378	Pimelic acid	Acid	0.06 (0.03)
37	20.12	1429	Suberic acid	Acid	1.00 (0.43)
39	22.26	1479	Azelaic acid	Acid	0.08 (0.05)
**Acids**					**27.76**
17	9.56	1173	Glycerol^a^	Alcohol	1.60 (0.48)
**Alcohols**					**1.60**
12	7.51	1113	16-Heptadecenal	Aldehyde	0.37 (0.18)
**Aldehydes**					**0.37**
13	8	1128	N-Trifluoroacetyl glycine	Amino acid	1.07 (0.62)
20	11.12	1213	Proline	Amino acid	25.33 (4.78)
24	12.99	1259	Homocysteine	Amino acid	0.94 (0.69)
26	13.58	1271	Unknown	Amino acid	0.01 (0)
31	16	1331	Pyroglutamic acid^a^	Amino acid	0.01 (0)
**Amino acids**					**27.36**
32	16.26	1337	Di-t-butyl-trimethylsilyloxytoluene	Aromatic	0.61 (0.34)
34	19.77	1420	Di-t-butyl-trimethylsilyloxytoluene isomer	Aromatic	1.03 (0.6)
36	19.92	1423	Phthalic acid	Aromatic	0.94 (0.87)
40	22.41	1483	Protocatechuic acid	Aromatic	0.09 (0.05)
**Aromatics**					**2.67**
6	5.4	1052	Acetal	Ether	0.85 (0.45)
**Ethers**					**0.85**
42	27.2	1601	Palmitic acid	Fatty acid	1.23 (0.12)
43	30.74	1693	Stearic acid	Fatty acid	2.93 (0.64)
**Fatty acids**					**4.16**
18	9.66	1174	Phosphoric acid^a^	Inorganic	26.69 (8.19)
35	19.83	1421	Unknown	Inorganic	4.99 (2.22)
**Inorganics**					**31.68**
44	36.18	1843	Monopalmitin	Mono glyceryl ether	0.04 (0.03)
45	39.01	1925	Monostearin	Mono glyceryl ether	0.01 (0)
**Mono glyceryl ethers**					**0.04**
7	5.71	1061	Hydroxylamine	Nitrogenous compound	3.14 (1.24)
9	6.78	1092	Cadaverine^a^	Nitrogenous compound	0.90 (0.16)
15	8.47	1141	Silanamine	Nitrogenous compound	1.34 (0.19)
**Nitrogenous compounds**					**5.37**
38	21.66	1465	Deoxy mannose	Sugar	0.01 (0)
41	22.92	1495	Myo-Inositol	Sugar	0.64 (0.32)
**Sugars**					**0.65**

Rt, Retention time; RI, Retention index. ^a^represents peaks confirmed by standards.

The relative percentage was determined using the chromatographic peak area. The total percentile levels for each class is bolded. Number in brackets represents std deviation of measurements.

Twenty-two Organic acids were detected accounting for 27.8% of the primary metabolites pool in the beer residue. IR analysis confirmed bands indicative of organic acids from their respective wave numbers: OH (acid), 3403–3423 cm^−1^; C=O (acid), 1706–1708 cm^−1^; and C-O (acid), 1319–1321 cm^−1^ and in accordance with GC-MS results (Fig. 3). Succinic acid was the predominant organic acid detected at 7.3%, which is in agreement with what is described in modern beer post-fermentation^14^. Succinic acid is usually found at low levels in barley, but it accumulates with yeast fermentation, as does lactic acid, which was detected at 3.1%^15^. Oxalic acid, which is commonly found in modern beer^16^ and is used as a marker for identifying ancient barely-beer fermentation^17,18^, was detected at 4.7%. Our analysis confirms its usage by the ancient Egyptians in their beer making^19^ as reported by Maksoud *et al*. (1994) for residues recovered from another predynastic installation site, HK24A, at Hierakonpolis^9^. Other minor detected organic acids were malonic, acetic, malic, and hexanoic acid, which are also found in modern beer^20,21^. In addition, two fatty acids also known in modern brews, *viz*., palmitic and stearic acids^22^, were also detected.

Amino acids accounted for 27.4%, with proline as the only major amino acid found at 25.3%, making it the second most abundant acid after phosphoric acid. Ancient Egyptians are believed to have used different fruits, such as dates or figs, in their brewing process to enhance beer flavor and quality^2^. Maksoud *et al*. (1994) identified dates and grapes in the Predynastic beer residues^9^, although further macrobotanical analysis has not confirmed this^23^. Nevertheless, the high abundance of proline may suggest that dates were added, as proline is enriched in this fruit^24,25^. Hydroxylamine was the main nitrogenous compound found in the beer residue at 3.1%; it is a by-product of ammonia oxidation and *S*-nitrosoglutathione detoxification, part of nitrosative stress response^26,27^.

### GC-MS analysis of volatiles in the beer residue

Volatile analysis using solid phase microextraction (SPME) coupled to GC-MS techniques was adopted in parallel for flavor profiling and resulted in the detection of 49 volatiles belonging to 9 chemical classes, with acids (56.3%), ketones/lactones (18.5%), and esters (12.1%) being the major ones alongside terpenes, nitrogenous compounds, aromatics, and aldehydes (Fig. 2C,D, Table 2). 12 Acids were detected, including nonoic, caproic, 2-methyl-butanoic, caprylic, pentanoic, heptanoic, benzoic, α-methylbutyric, and capric acids. Interestingly, caprylic acid, capric acid, heptanoic, and nonoic acids are part of the volatile acid mixture found in modern beer^28,29^. Caproic acid, a medium-chain carboxylic acids, is a product of fermentation by yeast via chain elongation^30^. These fatty acids are fermentation-products and signs of beer aging and yielding a rancid or goaty flavor^29^. γ-Nonalactone was the major lactone found at 7.6% in the beer residue specimens. It exhibits a coconut-like odor and has been detected as the most odiferous compound in American bourbon whisky and other modern beers^31–33^. Nine esters were identified with laurate and 2-methylpropanoate esters being the major ones. Laurate and 2-methylpropanoate esters have been reported in modern beer^20,33^. The only major terpene that was found in the beer residues was geranyl acetone at 2%, and it is also found in modern beer^34^.

**Table 2:** Relative percentage of compounds detected in beer residues from vats using SPME-GC-MS for volatiles analysis (n = 3).

Peak	Rt (min)	RI	Name	Class	Average (Standard Deviation)
2	6.43	873	2-Methyl-butanoic acid	Acid	7.26 (7.26)
3	6.79	819	*α*-Methylbutyric acid	Acid	1.41 (1.38)
4	7.07	905	Pentanoic acid	Acid	5.85 (5.85)
5	7.33	919	Pentanoic acid isomer	Acid	2.80 (2.74)
7	8.18	969	Pyroterebic acid	Acid	0.17 (0.17)
8	8.73	1001	Caproic acid^a^	Acid	8.31 (8.07)
13	10.07	1086	Unknown	Acid	8.25 (0.16)
14	10.2	1095	*n*-Heptanoic acid^a^	Acid	2.91 (2.81)
18	11.28	1170	Caprylic acid	Acid	6.71 (0.9)
20	11.57	1180	Benzoic acid	Acid	1.44 (0.46)
22	12.49	1259	Nonoic acid	Acid	10.84 (6.76)
29	13.61	1348	*n*-Capric acid^a^	Acid	0.39 (0.23)
**Acids**					**56.31**
1	5.9	848	3-Hexen-1-ol^a^	Alcohol	0.25 (0.25)
23	12.52	1261	Unknown	Alcohol	0.83 (0.27)
44	17.6	1637	Unknown	Alcohol	0.24 (0.17)
**Alcohols**					**1.31**
12	9.97	1079	Nonanal^a^	Aldehyde	0.17 (0.17)
19	11.39	1177	Decanal^a^	Aldehyde	0.38 (0.36)
**Aldehydes**					**0.55**
6	7.81	947	Benzaldehyde^a^	Aromatic	0.25 (0.25)
10	9.53	1052	Acetophenone	Aromatic	0.85 (0.82)
15	10.95	1147	o-Acetylphenol	Aromatic	1.00 (0.98)
**Aromatics**					**2.090**
25	13.11	1307	Glycerin triacetate	Ester	0.57 (0.46)
26	13.32	1337	1-(2-Hydroxy-1-methylethyl)-2,2-dimethylpropyl 2-methylpropanoate	Ester	1.06 (0.53)
28	13.56	1323	3-Hydroxy-2,4,4-trimethylpentyl 2-methylpropanoate	Ester	2.45 (1.8)
36	15.39	1499	Butyl maleate	Ester	0.02 (0.01)
39	17.15	1631	Hedione	Ester	0.81 (0.27)
40	16.08	1542	Propanoic acid, 2-methyl-, 1-(1,1-dimethylethyl)-2-methyl-1,3-propanediyl ester	Ester	0.81 (0.27)
41	16.13	1546	unknown hydrocarbon	Ester	0.06 (0.03)
47	20.28	1778	Isopropyl myristate	Ester	0.21 (0.13)
42	16.53	1573	Isopropyl laurate	Ester	6.16 (5.51)
**Esters**					**12.13**
34	14.87	1451	Pentadecane	Hydrocarbon	0.62 (0.23)
38	16.13	1599	Hexadecane	Hydrocarbon	0.62 (0.27)
45	17.71	1642	Unknown	Hydrocarbon	1.76 (1.2)
46	17.73	1643	Unknown	Hydrocarbon	0.59 (0.24)
**Hydrocarbons**					**3.59**
17	11.2	1164	2-Decanone	Ketone	0.24 (0.24)
9	9.39	1043	γ-Caprolactone	Lactone	0.55 (0.55)
16	10.83	1138	γ-Heptalactone	Lactone	0.20 (0.2)
21	12.25	1241	γ-Butylbutyrolactone	Lactone	4.59 (1.73)
27	13.52	1341	γ-Amylbutyrolactone	Lactone	7.56 (4.33)
33	14.76	1441	γ-Decanolactone	Lactone	0.98 (0.47)
37	16.08	1542	γ-Decalactone	Lactone	4.36 (3.48)
**Ketones/Lactones**					**18.46**
24	12.58	1266	Unknown	Nitrogenous compound	2.11 (1.5)
**Nitrogenous compounds**					**2.11**
30	13.72	1357	Unknown	Terpene	0.50 (0.31)
31	13.95	1375	Geranyl isobutyrate	Terpene	0.04 (0.02)
32	14.39	1411	Geranyl acetone	Terpene	2.10 (1.43)
35	14.94	1457	Aromandendrene	Terpene	0.12 (0.03)
43	17.48	1631	Unknown	Terpene	0.20 (0.11)
48	20.57	1794	Hexahydrofarnesyl acetone	Terpene	0.16 (0.07)
49	21.44	1878	Farnesyl acetone	Terpene	0.10 (0.03)
**Terpenes**					**3.20**
11	9.59	1056	Unknown	Unknown	0.27

Rt, Retention time; RI, Retention index. ^a^represents peaks confirmed by standards.

The relative percentage was determined using the chromatographic peak area. The total percentile levels for each class is bolded. Number in brackets represents std deviation of measurements.

Archaeo-botanical analyses of remains from various locations at predynastic Hierakonpolis indicate the cultivation of barley and emmer wheat, with emmer being the predominant crop^35^. Macrobotanical examination of the beer residues also suggested that emmer wheat was the major component in the beer produced at the site^5,9^. From the point of view of metabolites composition, barley and emmer wheat differ in the relative abundance of their constituents, such as their phenolics and acids, but they do not have any unique constituents that distinguish them^36^. Thus, it is hard to define what ratio of these grains was used in the beer. Numerous constituents of both were detected in the residue sample; however, suberic acid found at 1%, glycerol at 1.6%, and pyroglutamic acid < 1%, have a higher abundance in wheat than barley, lending support to the macrobotanical observations.

## Conclusion

The fermenting of grain-based foods is an ancient activity that goes back to nearly 13,000 years ago, even predating farming itself ^37^. The installation at locality HK11C of Hierakonpolis is the oldest dated brewery in Egypt and one of the earliest large-scale brewing sites in the world. As our analysis of the residues from its vats suggests, over time the ancient brewers developed ways to improve the quality, taste and durability of their product, as well as the technology to produce it in large quantities. Our detailed metabolites analysis using a mass spectrometric approach for profiling the beer residue confirms that brewing was conducted at the site and provides a detailed view of the chemical makeup of the ancient beer produced there. It has revealed various organic acids and yeast fermentation products that are the signature of beer, ancient or modern. Given that the evidence for fermentation was detected in the residue obtained from the interior of the vats, the fermentation process may have taken place in the same vats after the wort had cooled down. Our analysis also indicates that fruits, such as dates, may have been added to enhance sugar content and taste. Most significantly, our analysis suggests that the Predynastic Egyptians may have already recognized the enhanced preservation imparted by the addition of barley due to its enrichment in phosphoric acid. Such an understanding would have been critical to the development of this industry, allowing the early Egyptians to produce, bottle and distribute large amounts of beer in the facility at HK11C without fear of rapid spoilage.

## Methods

### Materials acquisition

Materials for the analysis were the residues recovered from vats 1 and 2 at locality HK11C. Two specimens from different places at each vat were collected for metabolites analyses. The 4 samples were transferred from the site to Cairo with the permission of the Egyptian Ministry of Antiquities. Botanical observation and the radiocarbon dating were carried out at the National Research Center and the French Institute for Oriental Archaeology at Cairo. Calibration of ^14^C age utilized IntCal 13 in the OxCal 4.3 program^38,39^.

### Chemicals and fibers

Chemicals including standards were purchased from Sigma (St. Louis, MO, USA). A 50/30 µm divinylbenzene/Carboxen on polydimethylsiloxane on a StableFlex fiber was used. This SPME fiber was purchased from Supelco (Oakville, ON, Canada).

### Analysis of silylated primary metabolites

To analyze non-volatile primary metabolites, we followed our previously described protocol in Farag *et al*. (2017)^40^. First, a 100-μL of 70% aqueous extract was prepared. This was achieved by extracting 100 mg of residue powder with 5 mL of 50% methanol with sonication for 30 min, which was followed by centrifugation at 12,000 *g* for 5 min to remove any debris. Then, the 70% aqueous extract was evaporated under nitrogen until dryness. For derivatization by silylation, a 150-μL of *N*-methyl-*N*-(trimethylsilyl)-trifluoroacetamide (MSTFA) was added to the dried extract and incubated for 45 min at 60 °C. The samples were equilibrated at 28 °C and subsequently analyzed using gas chromatography-mass spectrometry (GC-MS). A Trace GC Ultra Gas Chromatograph, coupled with a Thermo Scientific ISQ Single Quadrupole Mass Spectrometer (Thermo Scientific Corp., USA), was used for profiling of non-volatile silylated metabolites. Chromatographic separation was achieved on a Rtx-5MS column (30 m (length) × 0.25 mm (inner diameter), 0.25 μm film thickness) to analyze derivatized bear residual samples. Injections were performed in a split mode (1:15) and the gas chromatograph was operated as follows: injector temperature at 280 °C, column oven temperature at 80 °C for 2 min, followed by a program at a rate of 5 °C/min until 315 °C, and kept at 315 °C for 12 min; Helium carrier gas at rate 1 mL/min. The transfer line and ion-source temperatures were adjusted at 280 °C and 180 °C, respectively. The mass spectrometer was run in electron ionization mode at 70 eV. The scan range used was 50–650 m/z. Metabolites were identified using the procedure detailed in Farag and Wessjohann (2012)^41^. For metabolite identification, peaks were deconvoluted using AMDIS software and identified using the following approaches: retention indices (RI) relative to *n*-alkanes (C_6_–C_20_), mass spectrum matching to National Institute of Standards and Technology (NIST) and WILEY libraries, and available authentic standards. Relative percentages of metabolites were determined based on individual metabolite peak area relative to the sum of all identified metabolites peak areas.

### Volatiles isolation by SPME method

Headspace volatiles analysis using solid-phase microextraction (SPME) was adopted from Farag *et al*. (2015) with a few modifications^42^. Briefly, residue from vats was ground, and a 200-mg was placed in a 1.5-mL glass vial. (*Z*)-3-Hexenyl acetate, absent from specimens’ volatile organic compounds, was used as an internal standard. (*Z*)-3-Hexenyl acetate was dissolved in water and added at a concentration of 10 μg/vial. Then, vials were immediately capped and the SPME fibers were inserted into the headspace above the sample. Finally, vials were placed on a temperature-controlled tray at 50 °C for 30 min to achieve optimum adsorption. A system blank was run as a control. This blank sample was run from an empty vat containing no organic residue.

### Analysis of volatiles collected using SPME

SPME-isolated volatiles were subsequently analyzed using GC-MS. SPME fibers were desorbed at 210 °C for 1 min in the injection port of a Shimadzu Model GC-17A gas chromatograph interfaced with a Shimadzu model QP5000 mass spectrometer (Japan). Volatiles were separated on an Agilent J&W DB-5ms column, 30 m (length), 0.25 mm (inner diameter), 0.25 μm (film) (Agilent Technologies, Santa Clara, CA, USA). Injections were performed in the splitless mode for 30 s. The gas chromatograph was operated as follows: injector temperature at 220 °C, column oven temperature at 38 °C for 3 min, then programmed at a rate of 12 °C per min to 180 °C, kept at 180 °C for 5 min, finally ramped at a rate of 40 °C per min until 220 °C and kept there for 2 min; Helium carrier gas at 1 mL/min. The transfer line and ion-source temperatures were adjusted under analysis of silylated primary metabolites. The mass spectrometer was operated as previously described under analysis of silylated primary metabolites, except for the scan range, which was set at 40–500 m/z. Volatile metabolites were identified as previously described under analysis of silylated primary metabolites.

### IR analysis

Residue samples were weighed to be in a specific mass, then mixed with spectroscopic grade of dried potassium bromide, obtained from Specac (UK) to obtain proper weight/weight concentration of sample/diluent. Prepared pellets were placed in a Fourier-transform infrared spectrophotometer (Jasco FT/IR-6100, Japan). Spectra, for each sample, were recorded in the range of 4000–400 cm^−1^, scanning resolution of 4 cm^−1^, 256 single scans, and initial delay of 300 s. For each set of analysis, clear potassium bromide was used as a background.

### Abbreviations

BCE, Before Common Era; BP, Before Present; SPME, solid phase microextraction; GC, gas chromatography; MS, mass spectrometry; RI, retention index; MSTFA, N-methyl-N-(trimethylsilyl)-trifluoroacetamide; PCA, principal component analysis; HCA, hierarchical clustering analysis; NIST, National Institute of Standards and Technology; IS, internal standard; IR, infrared.

## Data Availability

All data are presented in the main text. Raw data is available at MetaboLights under MTBLS1302 study (www.ebi.ac.uk/metabolights/MTBLS1302)^43^.
